# Trimethylamine N-oxide (TMAO) in patients with subarachnoid hemorrhage: a prospective observational study

**DOI:** 10.1007/s00701-022-05485-3

**Published:** 2023-01-25

**Authors:** Julian Josef Emonds, Felix Arlt, Alexander Gaudl, Madlen Reinicke, Mitja Heinemann, Dirk Lindner, Sven Laudi, Uta Ceglarek, Jürgen Meixensberger

**Affiliations:** 1 Department of Neurosurgery, University of Leipzig Medical Center, Leipzig, Germany; 2 Institute of Laboratory Medicine, Clinical Chemistry and Molecular Diagnostics, University of Leipzig Medical Center, Leipzig, Germany; 3 Department of Anaesthesia and Intensive Medicine, University of Leipzig Medical Center, Leipzig, Germany

**Keywords:** Subarachnoid hemorrhage, Trimethylamine N-oxide, Intensive care units, Platelet aggregation, Gut-brain-axis, Blood-cerebrospinal fluid barrier disturbance

## Abstract

**Background:**

It is suspected that microbiome-derived trimethylamine N-oxide (TMAO) may enhance platelet responsiveness and accordingly be thrombophilic. The purpose of this prospective observational study is to evaluate TMAO in patients with subarachnoid hemorrhage (SAH) and compare it with a control group. A secondary aim was to investigate TMAO in the cerebrospinal fluid (CSF) from SAH patients. This should provide a better understanding of the role of TMAO in the pathogenesis of SAH and its thrombotic complications.

**Methods:**

The study included patients with diagnosed spontaneous SAH recruited after initial treatment on admission and patients with nerve, nerve root, or plexus disorders serving as controls. Blood samples were gathered from all patients at recruitment. Additionally, sampling of SAH patients in the intensive care unit continued daily for 14 days. The CSF was collected out of existing external ventricular drains whenever possible.

**Results:**

Thirty-four patients diagnosed with SAH, and 108 control patients participated in this study. Plasma TMAO levels at baseline were significantly lower in the SAH group (1.7 μmol/L) compared to the control group (2.9 μmol/L). TMAO was detectable in the CSF (0.4 μmol/L) and significantly lower than in plasma samples of the SAH group at baseline. Plasma and CSF TMAO levels correlated positively. The TMAO levels did not differ significantly during the observation period of 15 days.

**Conclusions:**

Although we assumed that patients with higher TMAO levels were at higher risk for SAH a priori, plasma TMAO levels were lower in patients with SAH compared with control subjects with nerve, nerve root, or plexus disorders on admission to the hospital. A characteristic pattern of plasma TMAO levels in patients with SAH was not found.

**Supplementary Information:**

The online version contains supplementary material available at 10.1007/s00701-022-05485-3.

## Introduction

Spontaneous subarachnoid hemorrhage (SAH) remains a major issue in patient care. It accounts for about 5% of all strokes with an incidence of around 6–9 per 100,000 per year worldwide [10, 12]. Younger people, with a mean age of around 55 years, are often affected by SAH [28]. A meta-analysis shows mortality of up to 66.7%, while up to 33% of the surviving patients are no longer able to return to work [26, 27]. Besides the initial bleeding, especially complications during the further course affect the outcome despite substantial progress in neurointensive care [16]. Delayed cerebral ischemia (DCI), for example, describes ischemia emerging 4 to 14 days after bleeding. Yet, the genesis of DCI is not well understood. In addition to vasospasm of the cerebral arteries, vascular injury caused by platelet aggregation and enhancing microvascular thrombus formation are discussed as possible mechanisms of DCI [44]. Such microvascular thrombi are found in patients with SAH and are presumed to be a part of DCI genesis. To date, no effective therapy has been elucidated against microvascular thrombus formation in SAH patients [5]. Hence, prediction and prevention are of utmost importance for both the management of SAH itself and its complications. The applicability of potential biomarkers such as trimethylamine N-oxide (TMAO) could contribute to this. The latter is known to be increased in cardiovascular risk patients and may be either a biomarker or even a mediator [17]. It is suspected that TMAO may enhance platelet responsiveness and accordingly be thrombophilic [53]. It has been associated with various diseases such as heart failure and stroke [35, 42, 43]. Contrary changes in TMAO levels have been reported in stroke patients, and the influence of TMAO on stroke patients has not yet been clarified. A separate observation of each form of a stroke may be reasonable. To the best of our knowledge, a possible impact of TMAO on SAH, in particular, has not been sufficiently explored. This study aimed to investigate the TMAO levels of patients in the acute phase of SAH and the course over the following days in the intensive care unit. The blood-CSF barrier (BCB) disturbance occurring in SAH prompted us to also investigate the presence of TMAO in cerebrospinal fluid (CSF) [25].

## Materials and methods

### Eligibility and study design

A prospective observational study including patients treated at the Department of Neurosurgery, University of Leipzig Medical Center (Leipzig, Germany), was performed from October 2018 to January 2020. The two study arms consisted of patients with diagnosed SAH treated at the intensive care unit (SAH group) and patients with nerve, nerve root, or plexus disorders treated at the normal unit serving as controls.

Patients with suspected non-traumatic SAH were screened for the SAH group. Patients with traumatic SAH or an age < 18 years were excluded from this study group. Patients with aneurysmal SAH and perimesencephalic SAH were considered as non-traumatic SAH. The SAH was confirmed by a neurosurgeon or neuroradiologist via cranial computed tomography followed by an intra-arterial selective digital subtraction angiography. Patients were treated either interventional with coiling or operative clipping of any aneurysms detected or conservatively with basic neuroprotective measures. Hydrocephalus was treated by external ventricular drainage if necessary, and all patients were monitored in the intensive care unit to control intracranial pressure (< 15 mmHg) and maintain sufficient cerebral perfusion pressure (> 70 mmHg). Patients do not routinely receive fibrinolytic substances, such as tranexamic acid, or the like. Nimodipine for the prophylaxis of delayed cerebral ischemia was started after initial management and at the earliest on day 1 after SAH. Transcranial Doppler examinations were performed daily to monitor blood flow velocities in the basal cerebral arteries. If a critical increase in blood flow velocities was observed, cranial computed tomography/computed tomography perfusion was performed to confirm a decrease in cerebral blood flow, and digital subtraction angiography was indicated. In the case of cerebral vasospasm being detected, inter alia, intraarterial treatment with nimodipine was started.

Patients with nerve, nerve root, or plexus disorders were screened for the control group. Patients with a history of proliferative processes, known damage to the central nervous system or an age < 18 years were excluded from this study group. The patient group that served as controls was selected from the patient population of the Department of Neurosurgery, University of Leipzig Medical Center (Leipzig, Germany), based on availability. We found no evidence for altered TMAO levels in this patient group [14, 40]. In addition, we did not expect any thrombotic events or cerebral abnormalities before treatment that could have affected the objective of the study.

### Study aims

The primary aim was to observe the difference in plasma TMAO levels between the SAH group at baseline and the control group.

Secondary aims were to observe the relationship between plasma TMAO and CSF TMAO, the association between plasma TMAO and the clinical severity of SAH, and the course of TMAO after SAH, and to identify possible factors influencing plasma TMAO levels.

### Sampling and data collection

Sampling for the SAH group started on the day of admission for suspected SAH (hereinafter referred to as “day 0 after SAH”) after the initial treatment. Whole blood samples were collected daily over 15 days into tubes containing ethylenediaminetetraacetic acid. Sampling stopped after 15 days, with the patient’s death or release from the intensive care unit. CSF was collected out of existing external ventricular drains where possible on admission and days 5 and 10 after SAH. Whole blood samples were collected from the control group only on admission into tubes containing ethylenediaminetetraacetic acid.

Additionally, hematocrit, hemoglobin, red blood cell count, platelet count, estimated glomerular filtration rate (eGFR), prothrombin time, and activated partial thromboplastin time were routinely measured for both patient groups. Therefore, samples were collected into tubes containing ethylenediaminetetraacetic acid, polyacrylate, or citrate, respectively.

Medical information was gathered anamnestically from the patients or their relatives or from existing medical records. The following patient monitoring data were gathered from the intensive care unit: the form of nutrition (sober, parenteral, enteral, oral), sedation status (sedated, not sedated), and intubation status (intubated, not intubated). The SAH was classified in all patients at baseline by a neurosurgical specialist according to the World Federation of Neurosurgical Societies (WFNS) classification for SAH [34]. Cranial computed tomography images obtained on admission were used by a neurosurgical specialist to grade patients according to the Fisher grading for SAH [13].

Albumin was measured in the CSF and plasma samples to calculate the CSF/plasma albumin ratio (*Q*_ALB_). The appearance of BCB disturbances was defined by an age-dependent upper limit of the *Q*_ALB_ < (4 + age /15) [33].

### Laboratory procedures

Measurements were performed at the Institute of Laboratory Medicine, Clinical Chemistry and Molecular Diagnostics, University of Leipzig Medical Center (Leipzig, Germany). Briefly, TMAO, betaine, carnitine, and choline plasma levels were determined by high-performance liquid chromatography-tandem mass spectrometry (LC–MS/MS). Samples were prepared by protein precipitation adding 90 μl of acetonitrile, including the internal standards, to 10 μl of the sample. After thorough mixing and centrifugation, 10 μl of the supernatant was diluted with 990 μl of eluent B. An amount of 5 μl of this mixture was used for liquid chromatography-tandem mass spectrometry analysis (a detailed method description can be found in Supplement, Expanded materials and methods). The method described for quantification of TMAO and its precursors has been used in previous studies [36], and recently a publication with the exact method description and validation has been published [8].

Hematocrit, hemoglobin, red blood cell count, and platelet count were measured on a Sysmex XN9000 analyzer (Sysmex Corporation, Kobe, Japan), according to the manufacturer’s instructions. The eGFR was estimated by measuring the serum creatinine enzymatically on the Cobas 8000 platform (Roche, Basel, Switzerland) using the Creatinine Plus assay (Roche, Basel, Switzerland) according to the manufacturer’s instructions. Prothrombin time was measured on the ACL top 700 platform (Instrumentation Laboratory, Bedford, USA) using the RecombiPlasTin 2G assay (Instrumentation Laboratory, Bedford, USA), according to the manufacturer’s instructions. The activated partial thromboplastin time was measured on the ACL top 700 platform (Instrumentation Laboratory, Bedford, USA) using the synthASil assay (Instrumentation Laboratory, Bedford, USA), according to the manufacturer’s instructions. Albumin was analyzed in plasma and CSF using a commercially available test kit on a Roche Cobas C 111 Clinical Chemistry analyzer (Roche Diagnostics, Mannheim, Germany).

### Statistical analysis

Continuous variables are presented as medians (interquartile range), unless specified otherwise, whereas categorical and dichotomous variables are presented as numbers and percentages. Variables were tested for normal distribution using the Shapiro–Wilk test. Differences between sampling times were calculated using the Wilcoxon test for non-normally distributed samples, whereas differences between the SAH group and the control group were calculated using the Mann–Whitney *U* test for non-normally distributed continuous variables and the Chi-square test for dichotomous variables. *p*-values of less than 0.05 were considered statistically significant. Correlations between parameters were calculated using Spearman’s rank test for non-normally distributed data. Statistical calculations were performed using IBM SPSS statistic 20 (Armonk, NY, USA). For post hoc power analysis, GPower 3.1.9.7 (Dusseldorf, Germany) was used.

## Results

### Baseline characteristics

We screened 54 patients with non-traumatic SAH and 135 patients with diagnosed nerve, nerve root, or plexus disorders. A total of 34 patients with diagnosed SAH or their legal representatives and 108 patients with nerve, nerve root, or plexus disorders agreed to recruitment.

Twenty-seven patients with SAH were treated interventionally and 7 patients conservatively. We gathered plasma samples from all patients on admission. External ventricular drains were laid in 17 patients with SAH, and CSF was collected on day 0 after SAH. Nine patients developed DCI during the observation period.

Characteristics at baseline were balanced between the two groups regarding sex, age, BMI, smoking status, and the prevalence of diabetes mellitus (Table 1). Hypertension occurred more often in the SAH group (79.4%) compared to the control group (52.8%). All SAH patients stated that they ate an omnivore diet.

**Table 1:** Demographic and baseline characteristics of the study population on admission

	SAH group (*n* = 34)	Control group (*n*= 108)	*p*-value	Ref
Men, *n* (%)	14 (41.2)	65 (60.2)	0.074 ^†^	
Age, yrs	53 (45–70)	57 (48–66)	0.586 ^†^	
BMI, kg/m^2^	26.1 (24.7–28.6)	28.1 (24.7–31.3)	0.094 ^†^	
Diabetes mellitus, *n* (%)	8 (23.5)	16 (14.8)	0.294 ^†^	
Hypertension, *n* (%)	27 (79.4)	57 (52.8)	0.009 ^†^	
Current smoker, *n* (%)	4 (11.8)	21 (19.4)	0.440 ^†^	
Deaths ^‖^, *n* (%)	4 (11.8)	0 (0)	0.003 ^†^	
WFNS classification, *n* (%)				
Grade I	7 (20.6)			
Grade II	7 (20.6)			
Grade III	4 (11.8)			
Grade VI	5 (14.7)			
Grade V	11 (32.4)			
Fisher grading, *n* (%)				
Grade I	0 (0)			
Grade II	5 (14.7)			
Grade III	6 (17.6)			
Grade VI	23 (67.6)			
Aneurysmatic SAH, *n* (%)	29 (85.3)			
Interventional care, *n* (%)	27 (79.4)			
DCI, *n* (%)	9 (26.5)			
Blood samples				
Samples, *n* (%)	34 (100)	108 (100)		
TMAO, μmol/L	1.7 (0.9–2.8)	2.9 (1.9–4.1)	< 0.001 ^†^	-
Betaine, μmol/L	20.3 (12.3–24.3)	31.5 (23.9–38.0)	< 0.001 ^†^	-
Carnitine, μmol/L	34.9 (28.8–43.0)	44.0 (37.9–51.2)	< 0.001 ^†^	-
Choline, μmol/L	4.5 (3.7–6.2)	6.7 (5.2–8.1)	< 0.001 ^†^	-
Hematocrit, %	0.32 (0.29–0.35)	0.41 (0.39–0.44)	< 0.001 ^†^	0.34–0.42
Hemoglobin, mmol/L	6.95 (6.03–7.53)	8.90 (8.30–9.53)	< 0.001 ^†^	7.30–10.10
RBC count, × 10^12^/L	3.58 (3.22–3.95)	4.70 (4.41–5.05)	< 0.001 ^†^	4.10–5.10
Platelet count, × 10^9^/L	230 (185–298)	245 (198–286)	0.629 ^†^	140–360
eGFR, mL/min/1.73m^2^	99 (84–110)	90 (75–104)	0.027 ^†^	> 90
Prothrombin time, %	92 (84–101)	105 (98–111)	< 0.001 ^†^	> 70
aPTT, s	26.0 (24.9–27.5)	29.0 (27.5–30.7)	< 0.001 ^†^	25.0–37.0
CSF samples				
Samples, *n* (%)	17 (50)			
TMAO, μmol/L	0.4 (0.2–0.9)		< 0.001 ^‡^	-
Betaine, μmol/L	3.4 (2.4–4.9)		< 0.001 ^‡^	-
Carnitine, μmol/L	1.4 (1.1–1.7)		< 0.001 ^‡^	-
Choline, μmol/L	2.4 (1.7–3.5)		< 0.001 ^‡^	-

Values are presented as median (interquartile range), unless otherwise stated. † Variables of the SAH group were compared with non-SAH serving as controls. ‡ Plasma samples were compared with CSF samples of the SAH group. ‖ Death during the observation period. *p* values of continuous and dichotomous variables were calculated using the Mann–Whitney *U* test, Fisher’s exact test, and Wilcoxon test, respectively. *aPTT*, activated partial thromboplastin time; *BMI*, body mass index; *CSF*, cerebrospinal fluid; *DCI*, delayed cerebral ischemia; *eGFR*, estimated glomerular filtration rate; *RBC*, red blood cell; *Ref*, reference value; *SAH*, subarachnoid hemorrhage; *TMAO*, trimethylamine N-oxide; *WFNS*, World Federation of Neurosurgical Societies

Hematocrit, hemoglobin, and red blood cell count on admission were significantly lower in the SAH group compared to the control group. Prothrombin time and activated partial thromboplastin time were significantly lower in the SAH group but within the reference range. The eGFR was higher in the SAH group but was above the reference threshold for both groups.

### TMAO and precursors at baseline

**Fig. 1:**
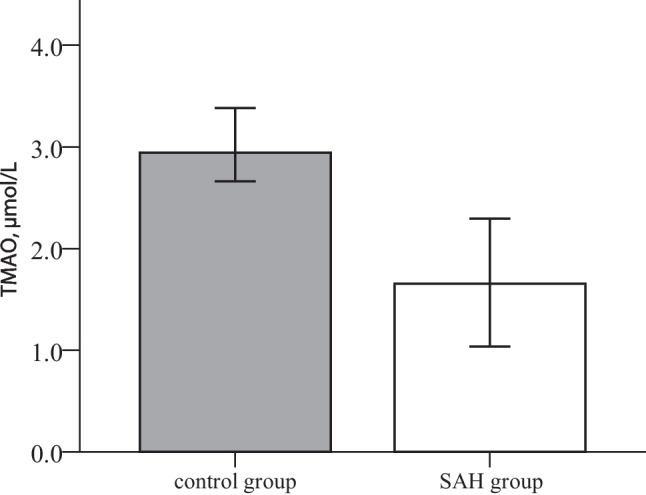
Differences in TMAO levels between SAH and control group at baseline. Values are presented as median with a 95% confidence interval. *p-*values were calculated using Mann–Whitney *U* test for non-normally distributed continuous variables. The SAH indicates subarachnoid hemorrhage and TMAO, trimethylamine N-oxide

Plasma TMAO levels were significantly lower in the SAH group at baseline compared to the control group (1.7 versus 2.9 μmol/L; Fig. 1). Post hoc power analysis for the Mann–Whitney U test yielded a power of 0.90 with an effect size of d = 0.659, alpha = 0.05, and the corresponding number of cases.

**Fig. 2:**
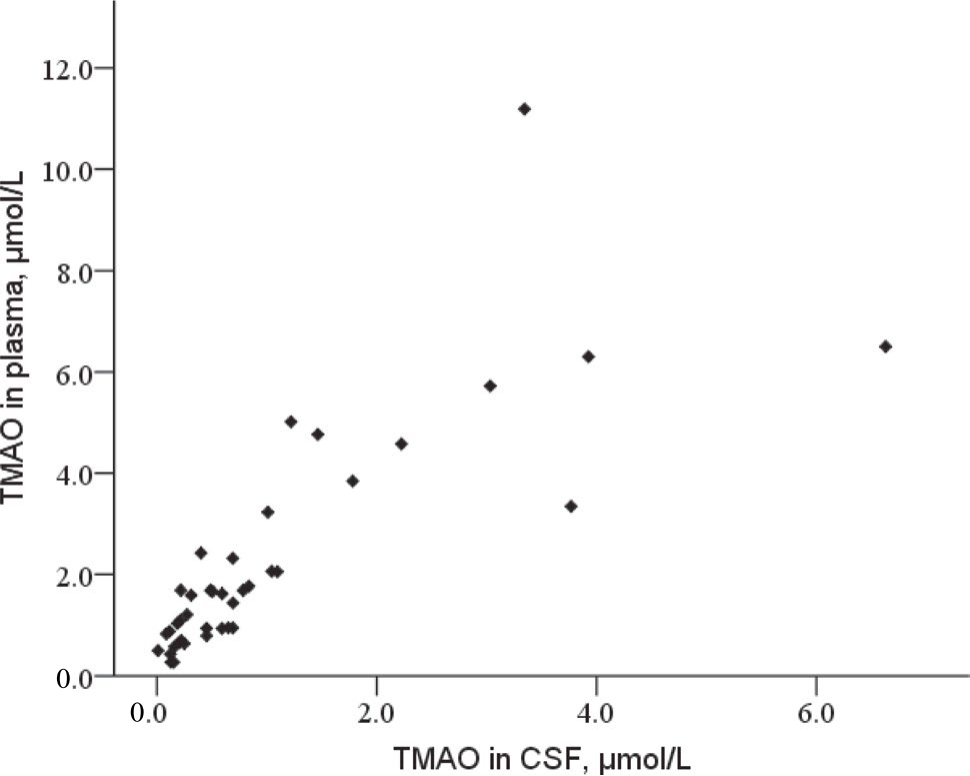
TMAO concentration in plasma and corresponding CSF sample in the SAH group at baseline. The SAH indicates a subarachnoid hemorrhage; CSF, cerebrospinal fluid; and TMAO, trimethylamine N-oxide

The CSF TMAO levels in the SAH group were fourfold lower than plasma TMAO levels at baseline (0.4 versus 1.7 μmol/L). Fifteen patients with SAH had a BCB disturbance defined by the Q_ALB_. The Q_ALB_ did not correlate significantly with CSF TMAO levels or the CSF/plasma TMAO ratio (Q_TMAO_). The subgroups divided by the appearance of a BCB disturbance did not differ significantly. Moreover, plasma TMAO levels correlated positively with the corresponding CSF TMAO levels (*p* < 0.001) (Fig. 2), whereas correlations between the CSF TMAO and its precursors in plasma samples were less high or not significant (Supplemental Fig. 1).

The TMAO precursors betaine, carnitine, and choline showed similar effects. Plasma levels were significantly lower in SAH patients compared to control patients. The CSF levels were significantly lower than plasma levels in the SAH group (betaine: 3.4 versus 20.3 μmol/L; carnitine: 1.4 versus 34.9 μmol/L; choline: 2.4 versus 4.5 μmol/L). Furthermore, CSF levels also correlated positively with corresponding plasma levels for all three precursors (Supplemental Fig. 1).

Patients who developed DCI had only significantly lower plasma carnitine levels than patients who did not develop DCI. Plasma and CSF TMAO levels did not differ between the DCI and non-DCI subgroups (Supplemental Table 1; Supplemental Fig. 2).

When evaluating subgroups defined by the WFNS classification, plasma TMAO levels did not differ significantly between these groups (Supplemental Table 2). Regrouping these subgroups into a good grade (WFNS classification 1–3) and a poor grade (WFNS classification 4–5), plasma TMAO levels tended to be lower in the poor grade subgroup, although the difference was not significant (Supplemental Table 3). No significant differences in plasma TMAO levels between the subgroups were found after dividing the SAH group by Fisher grading into 4 subgroups (Supplemental Table 4).

When comparing male and female patients across the groups of SAH and controls combined, males show significantly higher TMAO (2.8 versus 2.6 μmol/L), betaine (30.9 versus 21.7 μmol/L), carnitine (44.7 versus 37.9 μmol/L), and choline (6.6 versus 5.3 μmol/L) concentrations in plasma at baseline.

### Dynamic of TMAO after SAH

**Fig. 3:**
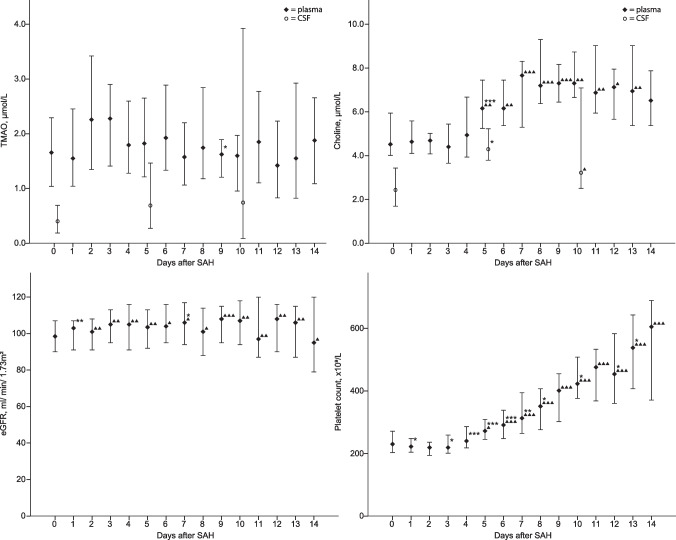
Changes in the median concentration of TMAO and choline in plasma and CSF samples and of eGFR and the platelet count in plasma samples of the SAH group over the observation period. Values are presented as median with a 95% confidence interval at each sampling time. *p*-values of continuous variables were calculated using the Wilcoxon test. *p ≤ 0.05 compared to the previous day; **p ≤ 0.01 compared to the previous day; ***p ≤ 0.001 compared to the previous day. ^▲^*p* ≤ 0.05 compared to day 0 after SAH; ^▲▲^*p* ≤ 0.01 compared to day 0 after SAH; and ^▲▲▲^*p* ≤ 0.001 compared to day 0 after SAH. The CSF indicates cerebrospinal fluid; eGFR, estimated glomerular filtration rate; SAH, subarachnoid hemorrhage; TMAO, trimethylamine N-oxide

Isolated failures in sampling occurred due to pronounced intensive care measures. The sample count decreased over the observation process because of discharge or death (Supplemental Table 5). A total of 390 blood samples were taken from all patients with SAH at all sampling times combined. Plasma TMAO levels did not vary significantly over 15 days after SAH (Fig. 3), and we did not observe a characteristic course. The eGFR varies significantly over the 15 days but was never below the reference threshold (> 90 mL/min/1.73 m^2^). The platelet count increased significantly after around 4 days and increased continuously until the end of the observation period. Plasma betaine levels increased in the days after SAH and become significantly higher compared to day 0 on the 7th day after SAH, while plasma carnitine and choline levels increased on the 5th day (Fig. 3, Supplemental Fig. 3). Since the absolute values of TMAO and its precursors were sometimes highly scattered, we observed the percentage changes of TMAO and its precursors, defining the value on day 0 after SAH as 100%. We did not find any considerably different courses compared to the absolute plasma levels (Supplemental Fig. 4). A total of 40 CSF samples were taken from 17 patients from the SAH group at all sampling times combined. The CSF TMAO levels did not vary significantly between days 0, 5, and 10 after SAH. Betaine, carnitine, and choline CSF levels increased significantly after day 0 (Fig. 3, Supplemental Fig. 3).

**Fig. 4:**
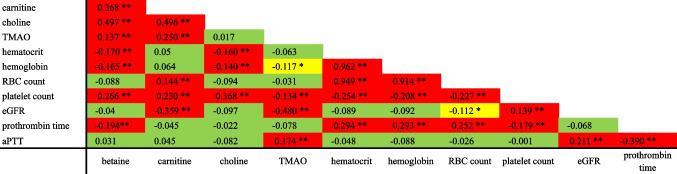
Correlation between TMAO, its precursors, and routine parameters of the SAH group over the observation period. Correlation between laboratory data over all 15 days combined. Correlation coefficients and significance were calculated using Spearman’s rho test. **Correlation is significant at the 0.01 level (2-tailed). *Correlation is significant at the 0.05 level (2-tailed). The TMAO indicates trimethylamine N-oxide; SAH, subarachnoid hemorrhage; RBC, red blood cell; eGFR, estimated glomerular filtration rate; aPTT, activated partial thromboplastin time

Plasma TMAO levels correlated significantly with hemoglobin, platelet count, eGFR, and activated partial thromboplastin time over all 15 visit times (Fig. 4). No significant differences in the plasma TMAO, betaine, carnitine, or choline levels between associated subgroups could be detected when comparing subgroups defined by the form of nutrition, sedation status, and intubation status on each day separately (Supplemental Fig. 5).

### TMAO and precursors at recovery or improvement

Plasma TMAO levels in the SAH group were significantly lower at recovery or the end of the observation period (improvement) than the control group at baseline (1.9 versus 2.9 μmol/L). Furthermore, plasma betaine levels were also significantly lower in the SAH group at this time point. In contrast, plasma choline levels were not significantly lower in the SAH group, and plasma carnitine levels were even not significantly higher compared to the control group (Supplemental Table 6).

## Discussion

Trimethylamine N-oxide is often associated with various diseases, such as atherosclerosis, diabetes, cancer, or stroke [14]. Since TMAO most probably affects the platelet responsiveness in humans, and thrombotic events may play a substantial role in the pathogenesis of SAH and its complications, investigations seem reasonable to clarify the role of TMAO, also as a biomarker, in these thrombotic processes [5, 53].

In our study, we found decreased plasma TMAO levels in patients with SAH compared to non-SAH controls. At recovery or the end of the observation period, we continued to find significantly lower plasma TMAO levels in the SAH group. Plasma TMAO levels of our control group were comparable to levels in a previous study with a central European cohort [23]. Additionally, hypertension occurred more often in the SAH group of our study. Hypertension is a typical risk factor for SAH but has also been associated with higher plasma TMAO levels previously [15, 41]. No other differences in demographics could be found. Mechanisms of action of TMAO include promotion of vascular inflammation, an important pathomechanism of cerebral aneurysms, and TMAO may even lead to disruption of the blood–brain barrier [3, 21, 51]. We hypothesized that patients with higher levels of TMAO would be at higher risk for SAH a priori because of the aforementioned mechanisms and that our patient group would therefore have higher TMAO levels on admission. In a hypertensive population, this effect should have been even greater.

Increased and decreased plasma TMAO levels have been reported for stroke patients, previously [11, 45, 49]. Schneider et al. could show that increased plasma TMAO levels on admission are followed by a decrease within the following 2 days [38]. This may be an explanation for contrary results. We could not find any characteristic course or significant changes in plasma TMAO levels after SAH, so TMAO levels remained decreased. Studies on TMAO in stroke patients are based mainly on ischemic stroke patients in mixed cohorts and should only be an indication for further research on TMAO in SAH patients. Risk factors, prediction, genesis, and management differ distinctively between ischemic and hemorrhagic stroke [1, 37, 46]. To the best of our knowledge, TMAO has not been investigated in patients with SAH. Hence, results should not only be interpreted based on recent findings. These differences could explain a lower plasma TMAO level in SAH patients compared to stroke patients in other trials.

One explanation for decreased TMAO levels in the SAH group could also be the so-called gut-brain axis, which refers to a “bidirectional communication network” between the nervous system and intestine [47]. We ingest TMAO mainly by eating fish, eggs, and meat products. Its precursors, e.g., betaine, carnitine, and choline, are metabolized by certain bacterial species of the gut microbiome. The product, trimethylamine (TMA), is incorporated via the intestine, transported to the liver, and metabolized by flavin-containing monooxygenases to TMAO [2, 6, 24, 32, 50]. Decreased plasma TMAO levels may result from acute brain damage and subsequent gut dysbiosis [4]. Patients with SAH typically experience sympathetic activation and a so-called catecholamine surge which could lead to downregulation of the immune system in the gut. Thereupon, the composition of the microbiome changes [18, 20]. Beneficial microbiota become increasingly deficient. The resulting decrease in TMAO levels has already been observed in patients with stroke or transient ischemic attack [49]. Apparently, the microspecies responsible for TMA production also perish or reduce production.

Worsened renal function is also reported to have a major impact on TMAO levels and explains higher plasma TMAO levels [31]. Although lower kidney function is reported in stroke patients, eGFR in the SAH group on admission was significantly higher (99 mL/min/1.73 m^2^) compared to the control group (90 mL/min/1.73 m^2^), and higher than the reference threshold of 90 mL/min/1.73 m^2^ in both groups [52]. The eGFR of the SAH group remained higher than the reference threshold for the entire observational period. The informational value of the eGFR for kidney function is distinctively lower, with an eGFR higher than 60 mL/min/1.73 m^2^ [39]. Accordingly, in our trial, we could hardly trace differences in plasma TMAO levels to the kidney function.

We did not find evidence in this trial for an association between the plasma TMAO and the clinical severity of SAH, but we did find hints of a negative correlation. Further investigations with higher numbers of cases will be necessary to prove such an association.

We expected higher TMAO levels in patients who develop DCI. DCI seems to be caused by an interplay of vascular inflammation and activation of the coagulation system, among other factors. This leads to vasospasm in vessels, from large arteries to arterioles, and microthrombus formation in small vessels [5, 44]. These effects could be exacerbated by the inflammatory and thrombotic potential of TMAO and could be more likely to lead to the development of DCI in patients with higher TMAO levels during the course of SAH. We did not find the expected difference between DCI and non-DCI cases and cannot support this hypothesis in our study. A reason for this could be the small number of 9 DCI cases.

Our results confirm the appearance of TMAO in the CSF and a correlation between plasma and CSF TMAO levels had already been reported. The correlation between the CSF TMAO and the plasma TMAO may be caused by BCB disturbances, but we did not find any correlation between *Q*_ALB_ and *Q*_TMAO_. BCB disorders typically occur after SAH, due to the destruction of endothelial cells and the disruption of tight junctions [25]. The BCB does not seem to be more permeable for TMAO, even if it is more permeable for albumin. Hence, we cannot support the conclusion made by a recent trial investigating CSF from 290 lumbar punctures [9]. The authors found a positive correlation between CSF TMAO and serum TMAO and an influence of the CSF/serum albumin ratio on the CSF/serum TMAO ratio, concluding that TMAO may cross the BCB via passive diffusion. Our results indicate that TMAO is transported over the BCB via active transport. CSF TMAO levels could potentially be a biomarker for or be associated with the genesis of central nervous diseases of any kind. Further investigations with a higher sample count are mandatory to confirm these observations.

The increase in precursors on the 5th to 7th day after SAH may be a sign of gut recovery. The missing increase of TMAO may be due to the length of the observation period. We also hypothesized that patients with thrombotic complications, such as DCI, would have an increase in TMAO after SAH. Although the pathogenesis of DCI can be attributed to vasospasm, the development of microvascular thrombi also seems to play an important role. It is well known that TMAO is associated with thrombotic events and atherosclerosis [17]. Zhu et al. revealed the possible promoting influence of TMAO on platelet responsiveness [53]. The pathologies remain largely unclear and mostly relate to cardiovascular disease. Unfortunately, there were too few cases of DCI in our study group to detect abnormalities in TMAO levels.

Interestingly, the platelet count started to increase simultaneously on the 5th day. A reactive thrombocytosis has already been described after SAH and could explain the increase [19]. A protective influence of carnitine on the platelet metabolism and function in stored platelet concentrates had also been found [7]. So far, the focus lies on the effect of TMAO and its precursors on platelet activity, but perhaps more attention should be paid to their effect on platelet metabolism, especially when evaluating the influence of TMAO on thrombus formation and DCI in SAH patients [5, 53].

Limitations of this study are the small count of SAH patients and the decreasing sample count during the observation period in the intensive care unit. Additionally, we were not able to determine the exact time point of hemorrhage and, therefore, could not integrate the time between the onset of symptoms and the first TMAO measurement into our analysis.

## Conclusions

Our results do not confirm our hypothesis that increased TMAO levels occur in patients with SAH or are related to its complications. Unexpectedly, plasma TMAO levels are decreased in SAH patients. The detection of TMAO in the CSF emphasizes a possible role of TMAO in central nervous processes. An impact of TMAO, or its absence, on patients with SAH or a role as a biomarker for the appearance of SAH and its complications would advance the understanding of this disease and could improve their management in the hospital. The exact effects and characteristics of TMAO in the human body should remain the focus of research. Further research with a larger number of SAH and DCI cases and different ethnicities is needed. Moreover, an analysis of the patients’ feces and the microorganisms contained should be added to the next investigations. The acquisition of TMAO into standardized biomarker patterns could, therefore, be a helpful instrument.

## Supplementary Information

Below is the link to the electronic supplementary material.

Supplementary file1 (PDF 1075 kb)

## Data Availability

The data presented in this study are available on request from the corresponding author. The data are not publicly available due to data privacy.
