# Supplementary material for: Trimethylamine N-oxide (TMAO) in patients with subarachnoid hemorrhage: a prospective observational study
Source: Acta Neurochir (Wien). 2023 Jan 25;165(5):1277–87. doi: 10.1007/s00701-022-05485-3 (PMC10140079; doi:10.1007/s00701-022-05485-3)
Supplement: Supplementary file 1 — Supplementary file1 (PDF 1075 kb) [file 701_2022_5485_MOESM1_ESM.pdf]

SUPPLEMENT

1 METHODS

Expanded Materials & Methods

Trimethylamine N-oxide (TMAO), betaine, carnitine and choline plasma levels were determined by high-performance liquid chromatography-tandem mass spectrometry. A HILIC-MS/MS method was established for the quantitation of betaine, carnitine, choline and TMAO according to published methods [50–53]. Within 3 hours of sampling, samples were centrifuged at 3500 rpm for 10 min and stored at -80 °C till analysis. The HILIC-MS/MS system consisted of a PAL HTS-xt autosampler from CTC Analytics (Zwingen, Switzerland), a Prominence UFLC system from Shimadzu (Duisburg, Germany) and a QTRAP® 6500 from SCIEX (Framingham, MA, USA). Chromatographic separation took place on a Kinetex® HILIC column (2.6 µm, 50 x 2.1 mm) from Phenomenex (Darmstadt, Germany). The mobile phase consisted of 15 mM ammonium formate buffer (pH = 3) in water/acetonitrile 90/10 v/v as eluent A and 15 mM ammonium formate buffer (pH = 3) in water/acetonitrile 10/90 v/v as eluent B. Separation was performed in gradient elution at a flow rate of 1.0 ml/min yielding a total run time of 5 min. Electrospray ionization was applied in a positive mode using the following mass transitions (m/z precursor/product) for quantitation: betaine 118/58, betaine-d9 127/68, carnitine 162/103, carnitine-d3 165/103, choline 104/60, choline-d9 113/69, TMAO 76/58 and TMAO-d9 85/66. Betaine, carnitine, choline, choline-d9, TMAO and TMAO-d9 were purchased from Sigma Aldrich (St. Louis, MO, USA). Betaine-d9 and carnitine-d3 were purchased from CDN Isotopes (Pointe-Claire, Quebec, Canada). Before analysis, the samples, calibrators and control samples were prepared by protein precipitation adding 90 µl of acetonitrile, including the internal standards, to 10 µl of the sample. After thorough mixing and centrifugation, 10 µl of the supernatant was diluted with 990 µl of eluent B. An amount of 5 µl of this mixture was used for LC-MS/MS analysis.

2 TABLES

Supplemental Table 1. Demographic and baseline characteristics of the SAH group divided into DCI and non-DCI cases.

	DCI	Non-DCI	p-value
n	9	25	
Men, n (%)	2 (22.2)	12 (48.0)	0.250
Age, yrs.	48 (36-64)	53 (47-72)	0.280
BMI, kg/m^2	25.7 (24.6-26.1)	26.9 (24.9-29.2)	0.188
Diabetes mellitus, n (%)	2 (22.2)	6 (24.0)	1.000
Hypertension, n (%)	8 (88.9)	19 (76.0)	0.644
Current smoker, n (%)	0 (0)	4 (16.0)	0.554
Aneurysmatic SAH, n (%)	9 (100.0)	20 (80.0)	0.293
Interventional care, n (%)	8 (88.9)	19 (76.0)	0.644
Blood samples			
Samples, n (%)	9 (100)	25 (100)	
TMAO, µmol/L	1.6 (0.7-4.9)	1.7 (0.8-2.6)	0.908
Betaine, µmol/L	15.5 (10.8-24.2)	20.6 (12.5-24.4)	0.618
Carnitine, µmol/L	28.6 (20.7-32.3)	38.1 (33.4-43.7)	0.007
Choline, µmol/L	4.0 (3.6-6.0)	4.5 (4.0-6.5)	0.489
Hematocrit, %	0.30 (0.27-0.32)	0.33 (0.30-0.35)	0.041
Hemoglobin, mmol/L	6.10 (5.60-6.95)	7.20 (6.30-7.60)	0.055
RBC count, x10^{12}/L	3.04 (2.80-3.67)	3.74 (3.47-4.04)	0.022
Platelet count, x10^9/L	249 (184-316)	222 (188-286)	0.539
eGFR, ml/min/1.73m^2	104 (83-116)	97 (84-109)	0.565
Prothrombin time, %	92 (72-100)	92 (85-101)	0.222
aPTT, s	26.9 (24.9-31.8)	26.0 (24.8-27.4)	0.414
CSF samples			
Samples, n (%)	7 (77.8)	10 (40)	
TMAO, µmol/L	0.5 (0.2-3.3)	0.3 (0.2-0.6)	0.536
Betaine, µmol/L	3.7 (2.4-5.4)	2.8 (2.3-4.8)	0.475
Carnitine, µmol/L	1.4 (1.2-1.6)	1.3 (0.9-1.9)	0.601
Choline, µmol/L	2.4 (1.4-3.4)	2.4 (1.7-3.6)	0.887

Demographic and baseline characteristics of the SAH group at admission divided into DCI and non-DCI cases. Values are presented as median (interquartile range) unless otherwise stated. DCI subgroup was formed from patients who developed DCI during the observation period. *P*

values of continuous and dichotomous variables were calculated using the Mann-Whitney U test and Fisher's exact test, respectively. The SAH indicates a subarachnoid hemorrhage; DCI, delayed cerebral ischemia; BMI, body mass index; TMAO, trimethylamine N-oxide; RBC, red blood cell; eGFR, estimated glomerular filtration rate; aPTT, activated partial thromboplastin time and CSF, cerebrospinal fluid.

Supplemental Table 2. Demographic and baseline characteristics of the SAH group divided by WFNS classification.

	WFNS I	WFNS II	WFNS III	WFNS IV	WFNS V
n	7	7	4	5	11
Men, n (%)	5 (71.4)	3 (42.9)	2 (50.0)	2 (40.0)	2 (18.2)
Age, yrs.	57 (45–69)	52 (42–63)	73 (56–83)	48 (46–65)	50 (34–64)
BMI, kg/m²	26.1 (22.0–30.4)	27.2 (26.1–30.4)	27.9 (27.0–31.9)	25.7 (24.2–26.9)	25.7 (24.1–26.1)
Diabetes mellitus, n (%)	3 (42.9)	1 (14.3)	2 (50.0)	0 (0.0)	2 (18.2)
Hypertension, n (%)	5 (71.4)	6 (85.7)	3 (75.0)	4 (80.0)	9 (81.8)
Current smoker, n (%)	0 (0.0)	1 (14.3)	1 (25.0)	1 (20.0)	1 (9.1)
Aneurysmatic SAH, n (%)	3 (42.9)	6 (85.7)	4 (100.0)	5 (100.0)	11 (100.0)
Interventional care, n (%)	3 (42.9)	6 (85.7)	2 (50.0)	5 (100.0)	11 (100.0)
Blood samples					
TMAO, µmol/L	2.1 (0.7–4.9)	2.1 (0.9–3.2)	2.4 (1.0–5.6)	1.0 (0.6–1.6)	1.6 (1.1–2.4)
Betaine, µmol/L	21.7 (15.3–23.3)	20.6 (15.5–26.2)	34.0 (18.1–53.3)	12.3 (8.2–24.1)	12.3 (9.9–23.4)
Carnitine, µmol/L	34.6 (32.4–42.9)	37.5 (29.9–60.4)	46.1 (38.8–52.9)	37.2 (24.4–43.7)	31.5 (23.6–38.8)
Choline, µmol/L	4.5 (3.1–6.1)	5.1 (4.2–6.6)	4.5 (3.4–11.1)	4.2 (3.3–5.7)	4.5 (3.6–6.1)
Hematocrit, %	0.33 (0.31–0.37)	0.32 (0.30–0.35)	0.34 (0.31–0.35)	0.33 (0.31–0.40)	0.27 (0.26–0.31)
Hemoglobin, mmol/L	7.30 (5.80–8.20)	7.10 (6.20–7.60)	7.15 (6.35–7.43)	7.40 (6.75–8.50)	5.80 (5.40–6.80)
RBC count, x10^{12}/L	3.75 (3.46–4.27)	3.57 (3.22–3.95)	3.95 (3.49–4.15)	3.74 (3.34–4.49)	3.04 (2.79–3.58)
Platelet count, x10^9/L	216 (190–298)	252 (203–315)	199 (184–225)	231 (201–330)	247 (183–299)
eGFR, ml/min/1.73m^2	95 (83–104)	102 (80–110)	88 (65–102)	109 (94–111)	95 (84–115)
Prothrombin time, %	93 (84–104)	92 (86–103)	92 (76–102)	93 (89–99)	89 (74–100)

aPTT, s	25.6 (24.5–39.0)	26.8 (25.0–27.4)	28.7 (24.8–30.4)	25.6 (23.5–26.0)	26.9 (25.8–27.4)

Demographic and baseline characteristics of the SAH group at admission divided by the WFNS classification. Values are presented as median (interquartile range) unless otherwise stated. Variables of the WFNS groups were compared with each other. *P* values of continuous and dichotomous variables were calculated using the Mann-Whitney U test and Fisher's exact test, respectively. No significant differences in plasma TMAO levels between the WFNS groups could be found. The SAH indicates a subarachnoid hemorrhage; WFNS, World Federation of Neurosurgical Societies; BMI, body mass index; TMAO, trimethylamine N-oxide; RBC, red blood cell; eGFR, estimated glomerular filtration rate and aPTT, activated partial thromboplastin time.

Supplemental Table 3. Demographic and baseline characteristics of the SAH group divided into good and poor grades.

	**good grade**	**poor grade**	**p-value**
n	18	16	
Men, n (%)	10 (55.6)	4 (25.0)	0.092
Age, yrs.	57 (50–71)	49 (41–63)	0.224
BMI, kg/m²	27.2 (26.1–30.4)	25.7 (24.2–26.1)	0.042
Diabetes mellitus, n (%)	6 (33.3)	2 (12.5)	0.233
Hypertension, n (%)	14 (77.8)	13 (81.3)	1.000
Current smoker, n (%)	2 (11.1)	2 (12.5)	1.000
Aneurysmatic SAH, n (%)	13 (72.2)	16 (100.0)	0.046
Interventional care, n (%)	11 (61.1)	16 (100.0)	0.008
Blood samples			
TMAO, µmol/L	2.1 (0.9–3.4)	1.5 (0.8–1.9)	0.266
Betaine, µmol/L	21.7 (16.0–25.1)	12.3 (9.7–23.3)	0.017
Carnitine, µmol/L	37.8 (32.8–49.8)	32.3 (23.7–39.8)	0.081
Choline, µmol/L	4.7 (4.0–6.5)	4.4 (3.6–6.1)	0.506
Hematocrit, %	0.33 (0.31–0.35)	0.30 (0.27–0.33)	0.075
Hemoglobin, mmol/L	7.15 (6.18–7.60)	6.55 (5.60–7.38)	0.251
RBC count, $x10^{12}$/L	3.81 (3.43–4.03)	3.45 (2.98–3.87)	0.088
Platelet count, $x10^{9}$/L	223 (189–278)	239 (183–301)	0.825
eGFR, ml/min/1.73m^2	98 (81–105)	101 (86–112)	0.443
Prothrombin time, %	92 (84–103)	91 (79–100)	0.231
aPTT, s	26.8 (24.8–28.0)	26.0 (24.9–27.4)	0.657

Demographic and baseline characteristics of the SAH group at admission divided by grading. Values are presented as median (interquartile range) unless otherwise stated. Variables of the WFNS groups were assigned to good grade (WFNS classification 1–3) or poor grade (WFNS classification 4–5) and compared with each other. *P* values of continuous and dichotomous variables were calculated using the Mann-Whitney U test and Fisher's exact test, respectively. The SAH indicates a subarachnoid hemorrhage; BMI, body mass index; TMAO, trimethylamine N-oxide; RBC, red blood cell; eGFR, estimated glomerular filtration rate; aPTT, activated partial thromboplastin time and WFNS, World Federation of Neurosurgical Societies.

Supplemental Table 4. Demographic and baseline characteristics of the SAH group divided by Fisher grading.

	Fisher I	Fisher II	Fisher III	Fisher IV
n	0	5	6	23
Men, n (%)	-	4 (80.0)	4 (66.7)	6 (26.1)
Age, yrs.	-	57 (51–67)	45 (38–49)	57 (48–72)
BMI, kg/m²	-	26.1 (23.9–29.7)	27.5 (22.5–55.5)	26.1 (25.4–27.4)
Diabetes mellitus, n (%)	-	2 (40.0)	1 (16.7)	5 (21.7)
Hypertension, n (%)	-	3 (60.0)	5 (83.3)	19 (82.6)
Current smoker, n (%)	-	0 (0.0)	1 (16.7)	3 (13.0)
Aneurysmatic SAH, n (%)	-	2 (40.0)	6 (100.0)	21 (91.3)
Interventional care, n (%)	-	2 (40.0)	6 (100.0)	19 (82.6)
Blood samples				
TMAO, µmol/L	-	2.1 (0.8–51.0)	1.2 (0.4–2.2)	1.7 (0.9–3.2)
Betaine, µmol/L	-	21.8 (20.2–23.7)	22.7 (18.1–28.2)	15.3 (10.7–25.0)
Carnitine, µmol/L	-	34.6 (32.7–39.0)	38.2 (26.8–49.7)	34.9 (23.9–44.0)
Choline, µmol/L	-	4.5 (3.3–5.3)	4.1 (3.7–5.5)	4.9 (3.6–6.7)
Hematocrit, %	-	0.33 (0.29–0.38)	0.35 (0.30–0.44)	0.31 (0.29–0.33)
Hemoglobin, mmol/L	-	7.30 (6.10–8.30)	7.60 (6.18–9.48)	6.60 (5.80–7.30)
RBC count, x10^{12}/L	-	3.65 (3.39–4.33)	4.28 (3.44–5.04)	3.51 (2.99–3.91)
Platelet count, x10^9/L	-	247 (215–453)	315 (197–340)	222 (184–252)
eGFR, ml/min/1.73m^2	-	95 (87–103)	110 (96–118)	95 (81–110)
Prothrombin time, %	-	93 (85–102)	86 (76–99)	92 (84–101)
aPTT, s	-	25.0 (24.2–27.0)	27.1 (25.1–38.7)	26.0 (25.0–27.6)

Demographic and baseline characteristics of the SAH group at admission divided by Fisher grading. Values are presented as median (interquartile range) unless otherwise stated. Variables of the Fisher groups were compared with each other. *P* values of continuous and dichotomous variables were calculated using the Mann-Whitney U test and Fisher's exact test, respectively. No significant differences in plasma TMAO levels between the Fisher groups could be found. The SAH indicates a subarachnoid hemorrhage; BMI, body mass index; TMAO, trimethylamine N-oxide; RBC, red blood cell; eGFR, estimated glomerular filtration rate; and aPTT, activated partial thromboplastin time.

Supplemental Table 5. Levels of TMAO and its precursors of the SAH group divided by days after SAH.

	D_0	D_1	D_2	D_3	D_4	D_5	D_6	D_7	D_8	D_9	D_{10}	D_{11}	D_{12}	D_{13}	D_{14}
Blood samples															
Samples, n	34	33	32	31	31	30	27	27	24	25	23	19	18	19	15
TMAO, μmol/L	1.7	1.6	2.3	2.3	1.8	1.8	1.9	1.6	1.7	1.6	1.6	1.9	1.4	1.6	1.9
	(0.9–2.8)	(0.9–3.1)	(1.1–3.7)	(1.0–3.3)	(1.0–3.5)	(1.1–3.5)	(1.3–3.1)	(1.0–2.5)	(1.0–2.8)	(1.0–2.2)	(0.8–2.0)	(1.1–2.8)	(0.8–2.3)	(0.8–2.9)	(1.1–2.7)
Betaine, μmol/L	20.3	19.1	19.5	19.4	17.6	21.1	21.9	22.1	23.6	25.0	22.0	25.1	23.9	21.3	22.3
	(12.3–24.3)	(12.6–24.3)	(12.8–23.3)	(14.2–22.4)	(13.1–22.4)	(16.6–26.5)	(18.2–27.9)	(16.2–27.7)	(17.6–35.0)	(20.7–34.5)	(17.3–33.5)	(21.6–31.4)	(15.6–30.5)	(17.7–32.9)	(18.0–28.3)
Carnitine, μmol/L	34.9	32.8	34.2	34.2	38.6	40.4	40.1	43.4	41.0	44.6	44.9	49.5	50.2	45.1	51.7
	(28.8–43.0)	(25.8–38.1)	(29.2–43.5)	(28.5–44.2)	(29.0–48.0)	(30.8–48.9)	(35.9–47.8)	(30.6–59.2)	(34.2–59.1)	(35.9–57.0)	(33.2–56.5)	(39.5–59.6)	(37.8–69.1)	(35.9–63.9)	(39.8–61.4)
Choline, μmol/L	4.5	4.6	4.7	4.4	4.9	6.2	6.2	7.7	7.2	7.3	7.3	6.9	7.1	6.9	6.5
	(3.7–6.2)	(3.9–5.8)	(4.1–5.1)	(3.5–6.2)	(3.9–6.8)	(4.8–8.3)	(5.2–7.7)	(5.1–8.4)	(6.3–9.4)	(6.1–9.0)	(6.5–8.7)	(5.9–9.0)	(5.5–8.0)	(5.4–9.0)	(5.4–7.9)
CSF samples															
Samples, n	17					15					8				
TMAO, μmol/L	0.4					0.7					0.7				
	(0.2–0.9)					(0.3–1.5)					(0.2–1.6)				
Betaine, μmol/L	3.4					6.1					7.4				
	(2.4–4.9)					(3.6–8.6)					(5.5–9.7)				
Carnitine, μmol/L	1.4					2.5					7.8				
	(1.1–1.7)					(1.8–4.8)					(3.6–8.7)				
Choline, μmol/L	2.4					4.3					3.2				
	(1.7–3.5)					(3.8–5.2)					(2.7–4.9)				

The TMAO and its precursors, betaine, carnitine and choline, in plasma and CSF at each sampling time over 15 days. Values are presented as median (interquartile range) unless otherwise stated. The SAH indicates a subarachnoid hemorrhage; TMAO, trimethylamine N-oxide; D_X, Day x after SAH and CSF, cerebrospinal fluid.

Supplemental Table 6. Levels of TMAO and its precursors of the SAH group at the end of the observation period compared to the control group.

	SAH group (n = 30)	control group (n = 108)	p-value
TMAO, µmol/L	1.9 (1.3-2.9)	2.9 (1.9–4.1)	0.009
Betaine, µmol/L	20.6 (16.3-26.1)	31.5 (23.9–38.0)	< 0.001
Carnitine, µmol/L	51.6 (31.5- 58.7)	44.0 (37.9–51.2)	0.123
Choline, µmol/L	6.1 (4.7-7.4)	6.7 (5.2–8.1)	0.114

TMAO, betaine, carnitine and choline plasma levels of the SAH group at the end of the observation period compared to the control group at admission. Values of the SAH group should represent the time point of recovery or improvement. Values are presented as median (interquartile range), unless otherwise stated. *P* values of continuous variables were calculated using the Mann-Whitney U test. The SAH indicates subarachnoid hemorrhage and TMAO, trimethylamine N-oxide.

3 FIGURES

Supplemental Figure 1. Correlation of TMAO and its precursors between CSF and plasma of the SAH group.

CSF \ Blood	betaine	carnitine	choline	TMAO
betaine	0.557*	0.210	0.599*	0.598*
carnitine	0.449	0.217	0.329	0.571*
choline	0.310	0.059	0.438	0.478
TMAO	0.342	0.194	0.475	0.850**

Correlation of the concentrations of TMAO and its precursors between plasma and corresponding CSF samples (n = 17) at day 0 after SAH. Correlation coefficients and significance were calculated using Spearman's rho test. ** Correlation is significant at the 0.01 level (2-tailed). * Correlation is significant at the 0.05 level (2-tailed). The SAH indicates a subarachnoid hemorrhage; TMAO, trimethylamine N-oxide and CSF, cerebrospinal fluid.

Supplemental Figure 2. Changes in the median concentration of TMAO and its precursors in plasma and CSF samples of the SAH group over the observation period divided by the occurrence of DCI.

A **B**

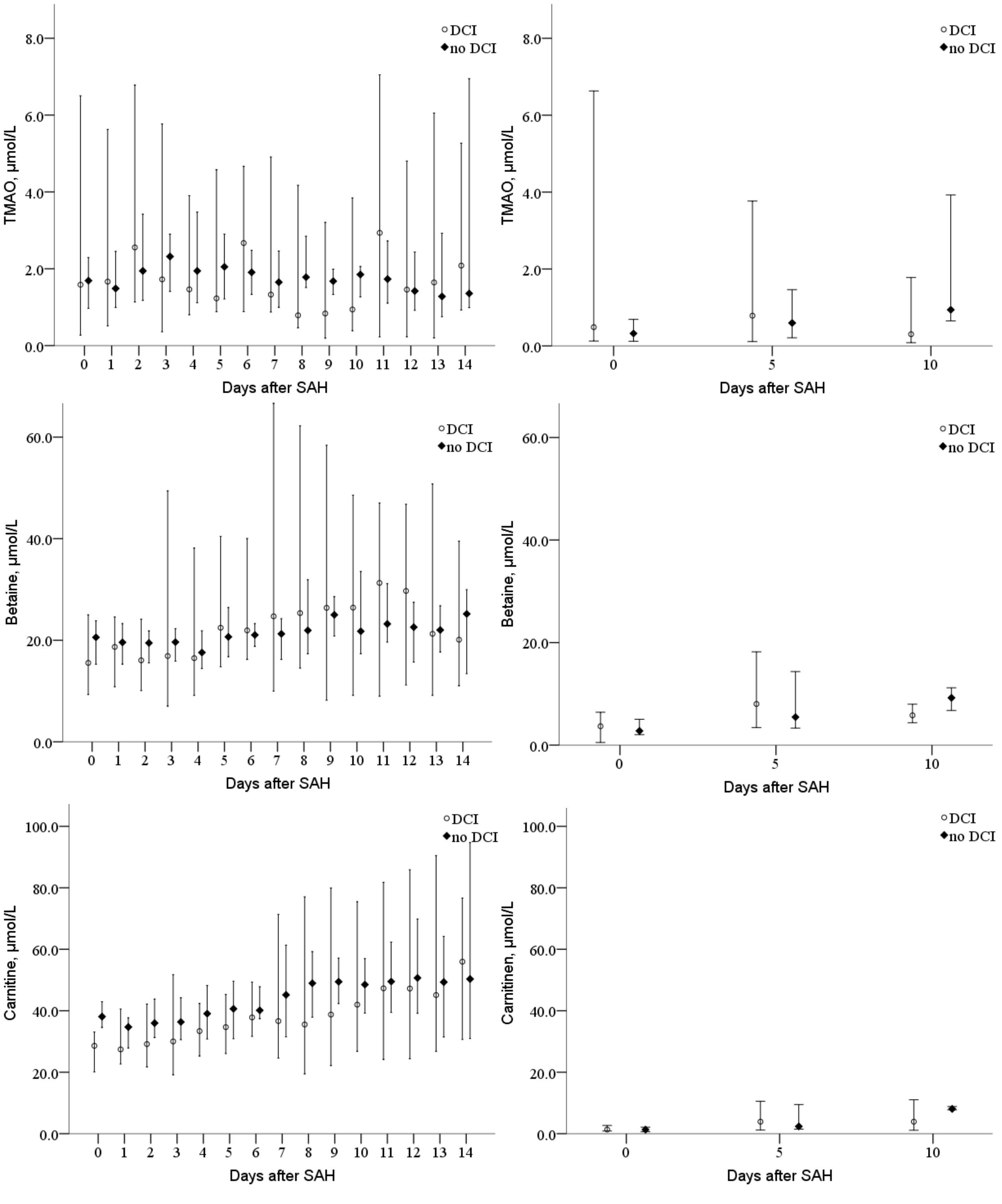

A **B**

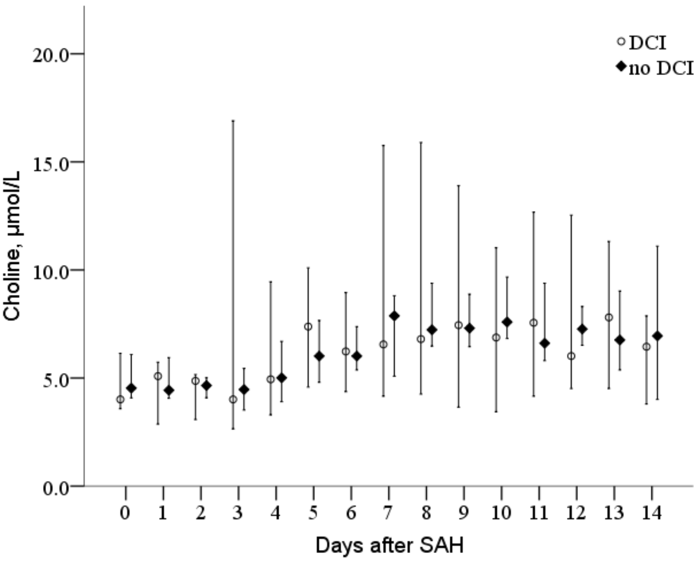

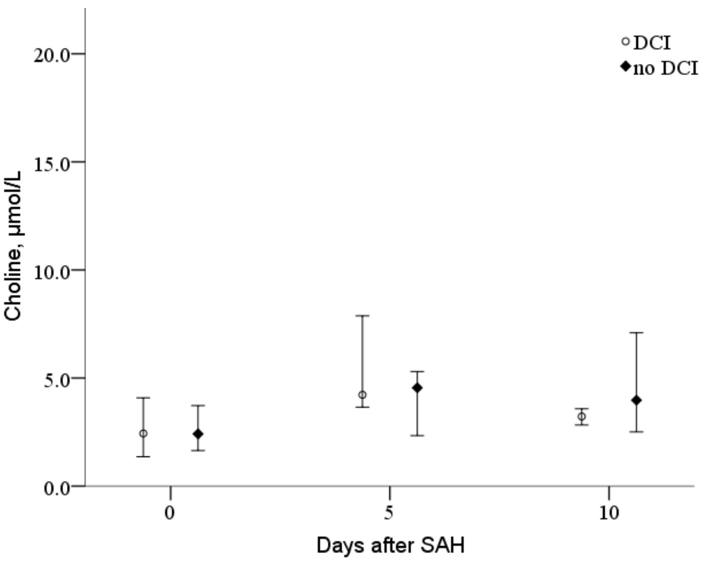

The course of TMAO and its precursors in plasma (A) and CSF samples (B) separated into cases with DCI (DCI) and without DCI (no DCI) over the 15 days after SAH. Values are presented as median with a 95 % confidence interval at each sampling time. The CSF indicates cerebrospinal fluid; DCI, delayed cerebral ischemia; SAH, subarachnoid hemorrhage and TMAO, trimethylamine N-oxide.

Supplemental Figure 3. Changes in the median concentration of routine parameters and TMAO precursors in plasma and CSF samples of the SAH group over the observation period.

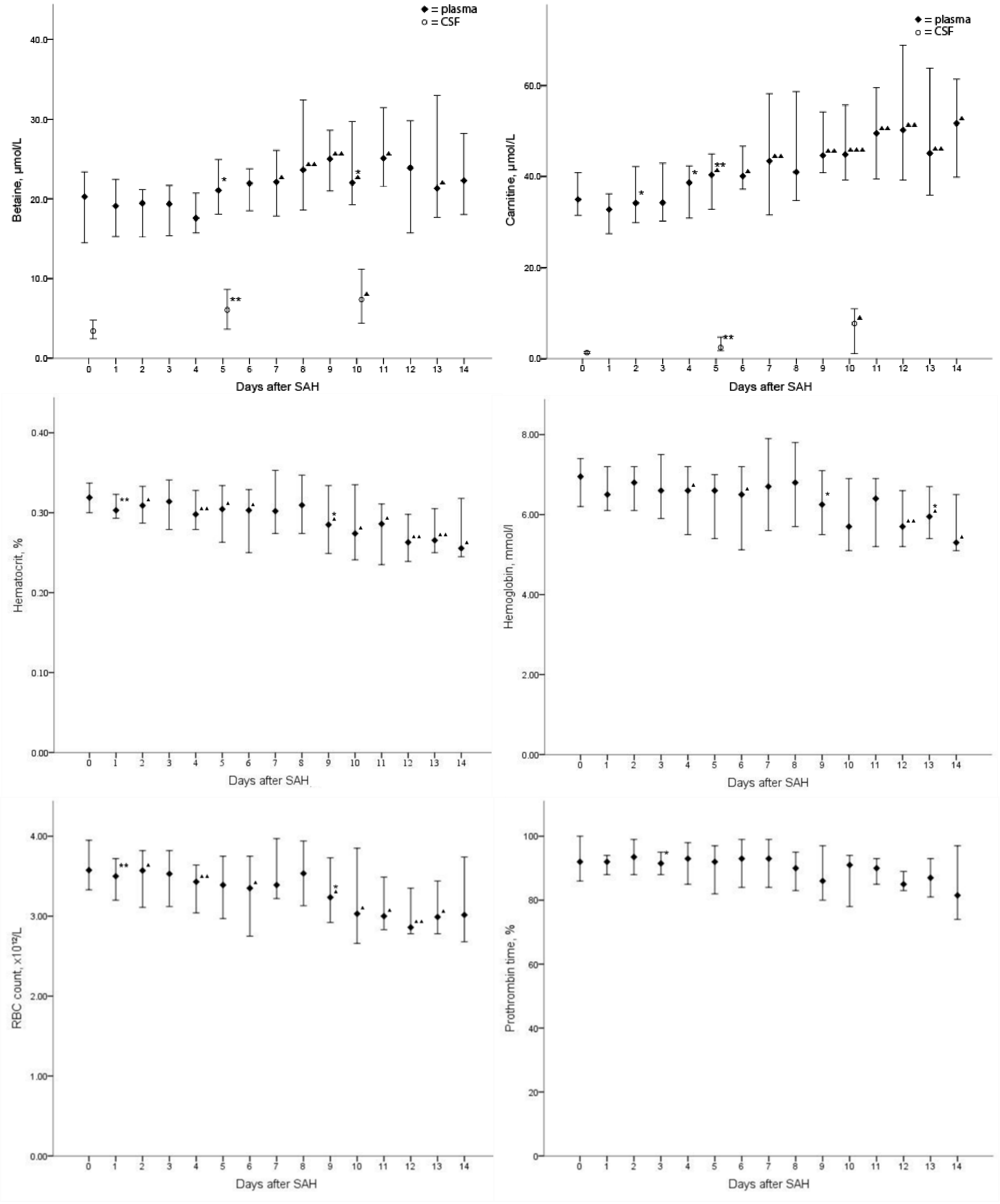

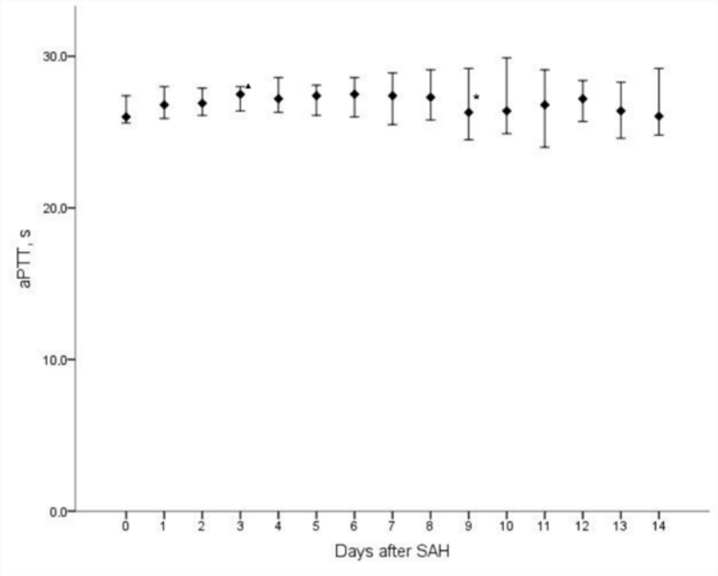

The course of routine parameters and TMAO precursors over 15 days after SAH in plasma and CSF (for betaine and carnitine) samples. Values are presented as median with a 95 % confidence interval at each sampling time. *P* values of continuous variables were calculated using the Wilcoxon test. * $p \leq 0.05$ compared to the previous day, ** $p \leq 0.01$ compared to the previous day, *** $p \leq 0.001$ compared to the previous day. ▲ $p \leq 0.05$ compared to day 0 after SAH, ▲▲ $p \leq 0.01$ compared to day 0 after SAH, and ▲▲▲ $p \leq 0.001$ compared to day 0 after SAH. The CSF indicates cerebrospinal fluid; SAH, subarachnoid hemorrhage; TMAO, trimethylamine N-oxide; RBC, red blood cell and aPTT, activated partial thromboplastin time.

Supplemental Figure 4. Relative changes in the median concentration of TMAO and its precursors in plasma samples of the SAH group over the observation period.

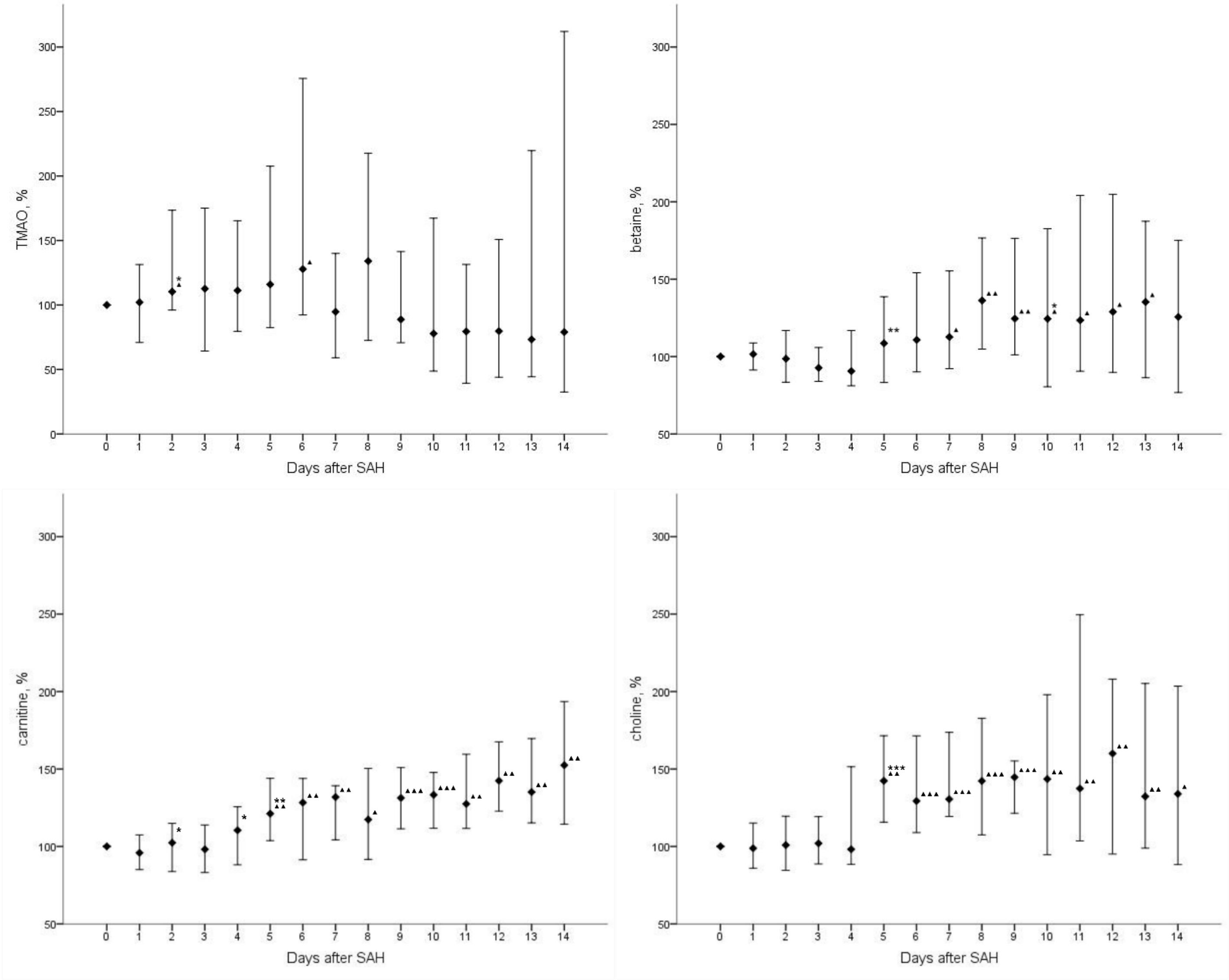

Relative changes of TMAO and its precursors in plasma samples over 15 days after SAH. Values are presented as median relative concentration compared to day 0 after SAH with a 95 % confidence interval at each sampling time. *P* values of continuous variables were calculated using the Wilcoxon test. * $p \leq 0.05$ compared to the previous day, ** $p \leq 0.01$ compared to the previous day, *** $p \leq 0.001$ compared to the previous day. ▲ $p \leq 0.05$ compared to day 0 after SAH, ▲▲ $p \leq 0.01$ compared to day 0 after SAH, and ▲▲▲ $p \leq 0.001$ compared to day 0 after SAH. The SAH indicates a subarachnoid hemorrhage and TMAO, trimethylamine N-oxide.

Supplemental Figure 5. Changes in the concentration of TMAO in plasma samples of the SAH group divided by the form of nutrition, sedation status and intubation status over the observation period.

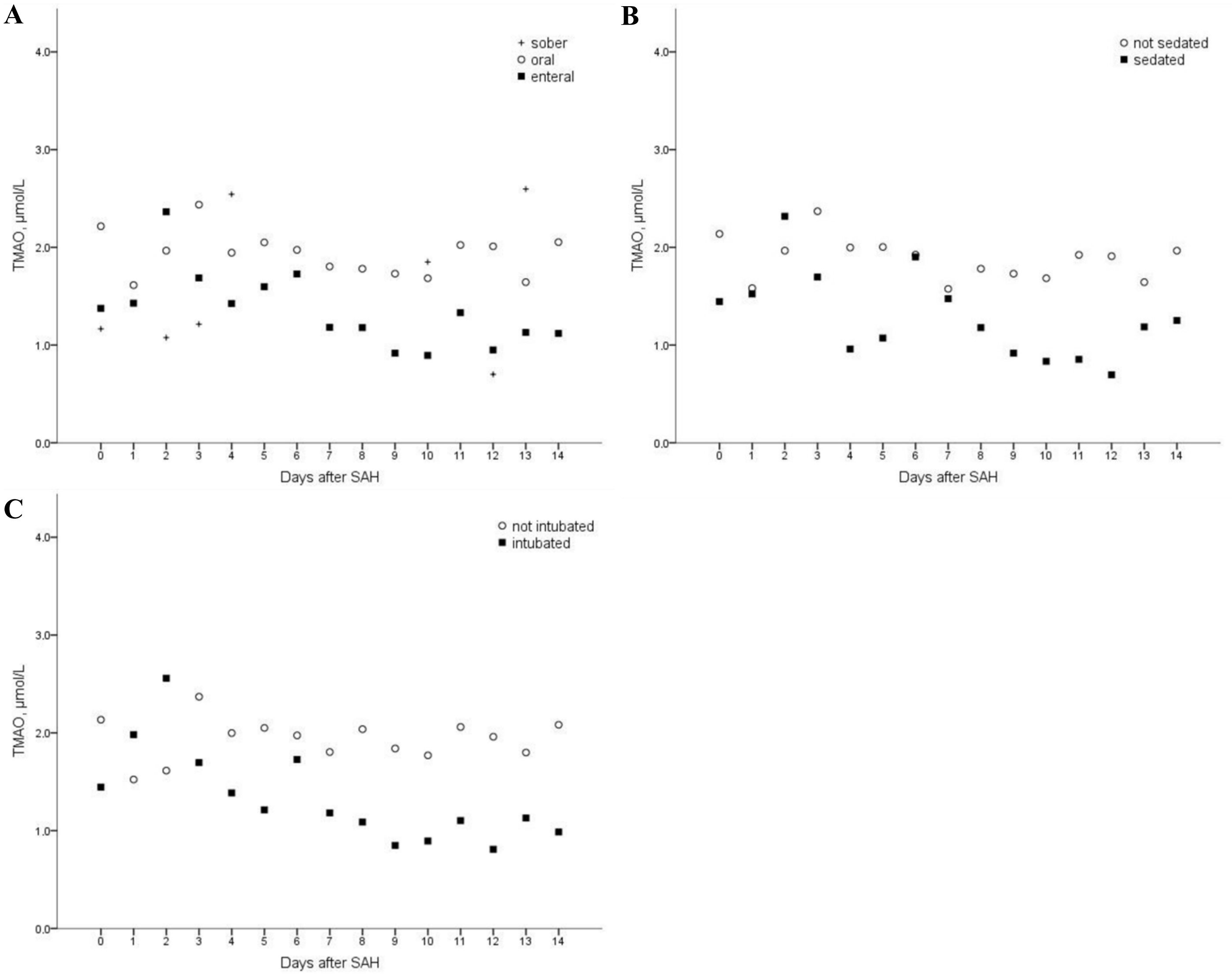

The course of TMAO in plasma samples over 15 days after SAH is taking possible influences on the concentration of TMAO into account. The sample count decreased from 34 to 15 patients over the observation process. **A** Samples divided by the form of nutrition. No significant differences between the subgroups sober (D_0: n = 2; D_{14}: n = 0), oral (D_0: n = 14; D_{14}: n = 9), and enteral (D_0: n = 18; D_{14}: n = 6) were found. **B** Patients divided by the sedation status. No significant differences between the subgroups sedated (D_0: n = 19; D_{14}: n = 3) and not sedated (D_0: n = 15; D_{14}: n = 12) were found. **C** Patients divided by the intubation status. No significant differences between the subgroups intubated (D_0: n = 17; D_{14}: n = 3) and not intubated (D_0: n = 17; D_{14}: n = 12) were found. Values are presented as medians at each sampling time. *P* values of continuous variables were calculated using the Mann-Whitney U test. The TMAO indicates trimethylamine N-oxide; SAH, subarachnoid hemorrhage and D_X, Day x after SAH.
